# Electroporation for the Treatment of Pancreatic Ductal Adenocarcinoma: A Systematic Review of Preclinical and Clinical Studies

**DOI:** 10.14309/ctg.0000000000000911

**Published:** 2025-08-29

**Authors:** Gabriel Marcellier, Theo Le Berre, Paul Rivallin, Marie Frenea-Robin, Frédéric Prat

**Affiliations:** 1 Endoscopy Unit, Beaujon Hospital, Clichy, France;; 2 Ecole Centrale de Lyon, INSA Lyon, Universite Claude Bernard Lyon 1, CNRS, Ampère, UMR5005, Ecully, France.

**Keywords:** electroporation, pancreatic cancer, pancreatic ductal adenocarcinoma, PDAC, electrochemotherapy, immunoelectroporation, irreversible electroporation

## Abstract

**INTRODUCTION:**

Pancreatic ductal adenocarcinoma (PDAC) is a highly aggressive malignancy with poor prognosis and limited treatment options. Electroporation-based therapies, such as electrochemotherapy (ECT) and irreversible electroporation (IRE), could be promising alternatives. ECT combines reversible electroporation with chemotherapy, enhancing intracellular drug uptake, while IRE leads to nonthermal tumor ablation. Both have been suggested as immunotherapy potentiators (electroimmunotherapy) in some tumor locations. We conducted a systematic review to evaluate the efficiency and safety of ECT, IRE, and immunoelectroporation in PDAC treatment.

**METHODS:**

We searched Medline, Embase, Cochrane, and Google-Scholar for ECT, IRE, and electroimmunotherapy following the Preferred Reporting Items for Systematic Reviews and Meta-Analyses guidelines. For ECT and electroimmunotherapy, regarding the scarcity of the data, we described independently each study protocol and results. For IRE, we collected protocol, efficiency, and safety data to provide a global analysis.

**RESULTS:**

Fifteen studies described the effects of ECT for PDAC treatment: Safety and efficiency were promising in both preclinical and human models. Thirty-eight clinical studies including 2,245 patients were analyzed for IRE, with patients mostly treated for locally advanced pancreatic cancer and a median overall survival of 17.2 months at the expanse of a 36% adverse event rate, half of which severe. Seven (preclinical and clinical) studies investigated electroimmunotherapy suggesting significant potentiation of immunotherapy in both preclinical and human models.

**DISCUSSION:**

In the largest systematic review to date regarding electroporation in PDAC treatment, analysis of study results plead against the use of IRE but highlight the potential benefits of ECT and electroimmunotherapy.

## Graphical abstract

**Figure:**
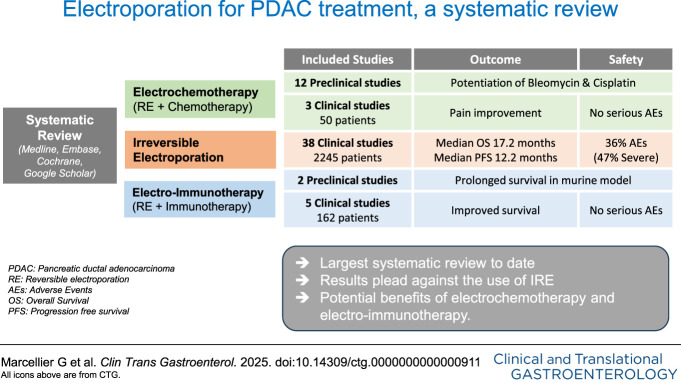

## INTRODUCTION

Pancreatic ductal adenocarcinoma (PDAC) represents one of the main causes of cancer mortality in western countries (1), with a 5-year survival rate of approximately 13% (American Cancer Society based on data from 2014 to 2020). Its constantly rising incidence combined with steady mortality rates despite therapeutic advances in oncology is expected to make it the second leading cause of cancer mortality by 2030 in some western countries (2) and a major public health issue worldwide (3).

The poor prognosis of PDAC is related to several factors. One is the absence of early diagnostic markers, which results in most cases being detected at locally advanced (stage III, locally advanced pancreatic cancer [LAPC]) or metastatic (stage IV) stages, when curative surgery is rarely feasible. Another is the presence of a dense, immunosuppressive tumor microenvironment (TME) that limits the effectiveness of most medical treatments (radiotherapy and chemotherapy) and acts as a barrier to immunotherapy (1).

The search for physical processes that disrupt the TME to improve chemosensitivity and/or allow immunomodulation could lead to survival improvement in PDAC.

Electroporation is based on the use of pulsed electric fields (PEFs), very high-voltage, short-duration current actuations that open up cell membrane nanopores, either temporarily (reversible electroporation [RE]) or permanently (irreversible electroporation [IRE]), depending on the duration and intensity of the current applied.

**Figure 1.**
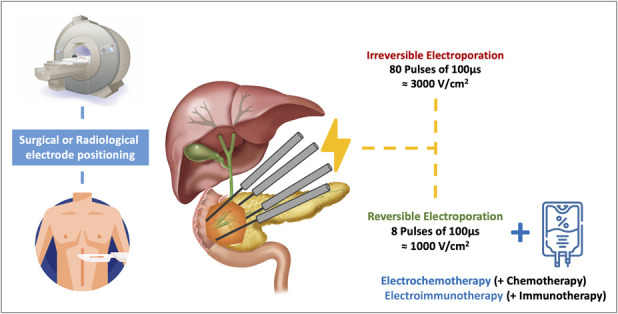
Electroporation for the treatment of human pancreatic ductal adenocarcinoma. Electrodes are positioned either surgically or under radiological control. 8–80 pulses of 1,000–3,000 V/cm^2^ are then applied to perform reversible or irreversible electroporation. The combination of reversible electroporation and chemotherapy or immunotherapy is, respectively, defined as electrochemotherapy or electroimmunotherapy.

In preclinical studies, a succession of 8 × 100 μs PEFs with an amplitude of 100–1,000 V/cm^2^ results in RE, while a succession of 80–100 PEFs around 3,000 V/cm^2^ exceeds the cell's adaptive capacity and results in IRE by electrolyte disturbance and cellular apoptosis (4,5) (Figure 1). Although RE does not cause cell death *per se*, it can enhance the cellular penetration of cytotoxic molecules. The use of RE to improve the effects of chemotherapy is thus called electrochemotherapy (ECT) (6). Effects of PEF on TME and the immune response to cancer invasion and metastasis have also been proven (electroimmunotherapy) (7). The exact mechanisms leading to cellular permeabilization under electrical fields remain an area of controversy, but electroporation has been used since 1968 for cellular transfer of DNA, pasteurization, and more recently cancer treatment with prostate, skin cancer, Kaposi sarcoma, among other recognized indications. The potential benefits of RE and IRE for the treatment of PDAC have logically been investigated. We conducted a systematic review of the clinical and preclinical literature on uses of electroporation for the treatment of PDAC.

## METHODS

### Screening methodology

We followed the Preferred Reporting Items for Systematic Reviews and Meta-Analyses 2020 guidelines for systematic reviews (8).

We searched articles regarding 3 major topics:

1. ECT in PDAC, defined by the use of RE to potentiate a cytotoxic agent. Regarding the scarcity of the human studies describing ECT for PDAC, we included preclinical works on *in vitro* and murine models.
2. IRE in PDAC. We focused on the human data describing the outcomes of IRE for PDAC treatment. Studies describing IRE for other neoplasm than PDAC, preclinical studies testing IRE on *in vitro* or *in vivo* models, feasibility studies, clinical protocols without any survival outcome (see below), and literature reviews were excluded from the systematic review. We also excluded case reports and case report compilations of less than 5 patients per category as judged too small to allow for survival calculations.
3. Electroimmunotherapy, defined by the use of electroporation to enhance immunotherapy. A very few studies are available on this topic, so we included preclinical and human studies implying reversible and/or IRE. Immunotherapy was defined as based on any drug aiming to improve the immune response against the tumor. We therefore present an exhaustive “state-of-the-art” review of preclinical and clinical studies.

Two physicians (F.P. and G.M.) from our unit went manually and independently through the below-mentioned databases. Keywords used for the research were pancreatic cancer, PDAC, electroporation, and ECT. Articles were screened from 1997 to 2024 across several databases (Medline, Cochrane Library, Embase, and Google Scholar). Databases were last checked on December 31, 2024.

### Data collection

For ECT and electroimmunotherapy, since the small number of studies did not allow statistical analysis, we reported individually the main available data from each preclinical or clinical study. We reported the type of drug used in association with electroporation and the electrical settings. If available, we also presented survival outcomes.

For IRE, due to the larger set of clinical data, only clinical studies were analyzed, with the following information being retrieved as available:

1. Patients and methodology: number of included patients, median tumor size, disease stage (III or IV), and median population age. The approach used for electrode positioning (surgical [SUR] or percutaneous [PC]), the prospective, retrospective, controlled design of the study, and the percentage of chemotherapy pretreated patients.
2. Outcomes: Studies had to provide at least one of the following “survival outcome” to be included: median overall survival (OS) from diagnosis or from IRE or progression-free survival (PFS) from IRE. Survival was expressed in months with interquartile range (IQR). If IQR were not provided in the publication, manual analysis of the Kaplan-Meier curves allowed calculation.
3. Safety: Median hospital stay duration was expressed in days. The number of patients who underwent an adverse event (AE) was noted for each study in association with the number of severe adverse events (SAEs) defined as Clavien-Dindo ≥3/5 (9). The main types of AEs described were also collected. In several studies, the number of patients experiencing AEs was not available (NA). We estimated that it was at least equal to the most frequently reported AEs (e.g., if 50 patients experienced fever and 45 pleural effusions, the number of patients with AEs was at least 50).

### Statistical analysis

For ECT and electroimmunotherapy, the scarcity of the available literature did not allow us to provide pooled statistical analysis. For IRE, statistical analysis was performed with the use of R Software (version 4.4.2). Since several data were NA, calculations were realized on the subpopulation where the analyzed data were present. For each analysis, we provided the size of the subpopulation and the percentage of the entire population it represents N (%).

We calculated weighted means for age, tumor size, hospital stays, and AEs/SAEs rates. The association between the AEs/SAEs percentages and the SUR or PC approaches was calculated with the Fischer tests.

For survival outcomes (OS and PFS), we calculated a weighted median in addition to the weighted mean to take the wide range of variables dispersion into consideration. We used the IQR to provide a boxplot of the survival outcomes for each study.

## RESULTS

### Literature screening

**Figure 2.**
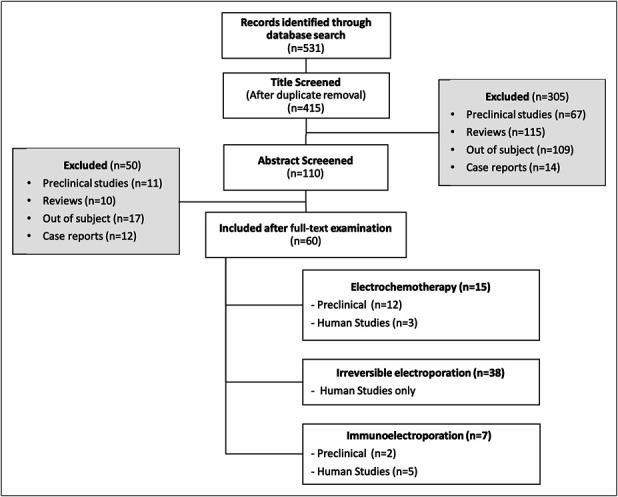
PRISMA flowchart of the systematic review. PRISMA, Preferred Reporting Items for Systematic Reviews and Meta-Analyses

The results of the systematic review are displayed in Figure 2. From the 415 studies identified through database research after duplicate removal, 60 were included in our review. Studies were excluded because they were either preclinical IRE studies (n = 73), because they were reviews or articles bringing no additional data (generalities about EP, letters, comments, study protocols, etc.) (n = 140), or because they were case reports (n = 24). Finally, some studies were excluded because being out of the topic (n = 118). Fifteen studies treated from ECT, 38 from IRE, and 7 form immune-electroporation.

### Electrochemotherapy

ECT relies on the ability of cells to open up membrane nanopores under the effect of PEFs. This allows chemical agents to which the cell is normally impervious to penetrate it during a few minutes (10). ECT application to control subcutaneous skin metastasis progression has been widely described in the European Standard Operating Procedures of Electrochemotherapy (ESOPE) protocol (5), which is worth being cited because most of the following works use the same electrical parameters (8 × 100 μs pulses at 1,000 V/cm—at a 5 kHz frequency) and cytotoxic agent, namely bleomycin. Although clinical literature is scarce regarding ECT for PDAC, several studies have reported potential benefits of ECT at several scales in preclinical PDAC models. Table 1 summarizes these different trials, their experimental settings, and their outcomes.

**Table 1.** Electrochemotherapy, preclinical trials

References	Model description	Drugs and electrical settings	Outcomes
*In vitro* studies			
Jaroszeski et al (11)	Several cell lines PDAC cell lines were cultured Capan-2 human pancreatic cells	Different voltages (500–4,000 V/cm) For PDAC cells, electric fields of 900 V/cm resulted in 90% cell survival, higher tensions tended to induce IRE Several drugs tested with calculation of the IC50	Ratios between IC50 with and without ECT were 700 for bleomycin, 12 for cisplatin, 2 for cytarabine, and 1.6 for carboplatin Ratios were ≈1 for the other drugs tested (vincristine, vinblastine, and mitomycin C)
Girelli et al (12)	PANC1 and MiaPaCa2 human PDAC cells	ESOPE protocol (8 × 100 μs pulses at 1,000 V/cm) Bleomycin and cisplatin	Without chemotherapy, RE induced 4% cellular death and permeabilization of 90% of PANC1 cells and 75% of MiaPaCa2 cells ECT lowered IC50 for PANC1 cells from 100 μM to 0.59 μM (169 ratio) for bleomycin 23 μM to 8 μM for cisplatin (3 ratio) ECT lowered IC50 for MiaPaCa2 cells from 3.5 μM to 0.2 μM (17.5 ratio) for bleomycin 30 μM to 23 μM (1.3 ratio) for cisplatin
Fernandes et al (13)	Human PANC1 cells Murine Pan02 cells	ESOPE protocol (8 × 100 μs pulses at 1,000 V/cm)	ECT significantly increased the lethality of bleomycin, cisplatin, and oxaliplatin on both PANC1 and Pan02 cells The death-related mechanism was necroptotic rather than apoptotic (like what gentamycin induces). Necroptosis induces inflammation and could then promote antitumoral immune response, explaining the abscopal effect
Bosnjak et al (14)	BxPC-3 human pancreatic cells	PEFs were delivered at 1,300 V/cm and 100 μs with a frequency of 1 Hz Bleomycin, cisplatin, and a tyrosine-kinase inhibitor (sunitinib) were tested	ECT potentialized the effects of bleomycin and cisplatin ECT with bleomycin was more effective than ECT with cisplatin Combination of ECT + bleomycin + sunitinib offered improved results compared with ECT + bleomycin alone. This synergistic effect was not identified for cisplatin
Michel et al (15)	Several cell lines derived from pulmonary metastasis of PDAC EPP85-181P (sensitive to daunorubicin) EPP85-181RDB (resistant to daunorubicin)	Cisplatin ESOPE protocol (8 × 100 μs pulses at 1,000 V/cm)	On the pulmonary metastasis cell lines, ECT improved the efficiency of cisplatin, even if the cells were cisplatin-resistant before ECT
*In vivo* studies			
Girelli et al (12)	Non pathological rabbit's pancreas	ESOPE protocol for PEFs No chemotherapy	Transaminase and amylase levels were not significantly modified, and all rabbits survived procedures Histologically, a strong inflammatory reaction was observed in the following days, but a complete recovery was noted at day 30
Dezman et al (16)	Electroporation on porcine pancreas 5 pigs received bleomycin-ECT 3 pigs received only PEFs	Surgical (laparotomy) electrode positioning in the pancreatic head and tail ESOPE protocol Animals sacrificed at day 7	2 pigs presented a transitory increase in pancreatic enzymes levels No sign of acute pancreatitis was observed on CT No thrombosis, no fistula, and no damage of the duodenum were identified. In one of the pigs, an ablation zone in the pancreatic head surrounded the main pancreatic duct while leaving the latter undamaged
Stupan et al (17)	Electroporation on porcine blood vessels 3 pigs received only PEFs 12 pigs received PEFs + bleomycin (3 sacrificed at day 7-14-28)	PEFs delivered on several locations and vascular surgical anastomosis (portal vein, inferior vena cava) PEF: 1,040 V for 20 μs	CT scans found no vascular thrombosis despite ECT but some anastomoses were strictured (possibly because of technical difficulties during procedures) Histological findings highlighted a vasculitis on the anastomosis with microthrombi. Mild inflammation and fibrosis were detected, and collagen fibers were intact. These reactions tend to heal over time at D14 and D28
Dev et al (18)	Human PANC3 cells were implanted subcutaneously on nude mice	Bleomycin ECT 6 needles 6 pulses of 99 μs at 1,340 V	Mice treated with ECT had tumor size decrease, while size increased in mice treated with bleomycin alone
Nanda et al (19)	Human cells implanted subcutaneously in nude mice	ECT with bleomycin, mitomycin, and carboplatin Electrical settings NA	Neither PEFs alone nor drugs alone were effective ECT + bleomycin was effective and more effective than ECT mitomycin and ECT carboplatin
Jaroszeski et al 1999 (20)	Hamsters with PDAC PC-1 cells orthotopic and subcutaneously injected	Bleomycin Electrical settings NA	No response for drug or PEF alone 100% total response for ECT in subcutaneous implantation 25% total response for orthotopic tumors
Dev et al (21)	Human PDAC cells subcutaneously injected in nude mice	ECT with bleomycin, mitomycin, and carboplatin 6 needles 6 pulses of 100 μs at 1,130 V at 4 Hz	Complete response rate at day 89 75% with bleomycin ECT 22% with mitomycin ECT 12.5% with carboplatin ECT

ECT, electrochemotherapy; IC50, 50% inhibitor concentration; PDAC, pancreatic ductal adenocarcinoma; PEF, pulsed electric field.

*In vitro* studies highlight the increased efficiency of bleomycin and cisplatin in PDAC cells, with bleomycin being the strongest potentiator. The ESOPE protocol was used in most study protocols. Innovative combination such as sunitinib could improve the cytotoxicity of bleomycin.

*In vivo* studies show that ECT seems to be safe when performed on large animals such as pigs. Its nonthermal effects reduce the number of complications compared with thermal techniques such as radiofrequency ablation (RFA) (22). Some data on electrical settings were NA. ECT preserves collagen membranes, leaving blood vessels and biliary/pancreatic ducts relatively undamaged (23). This has also been confirmed on humans with a 2017 study describing treatment of vascular portal metastasis of liver cancers with bleomycin ECT. In this study (6 patients), the thrombus seemed efficiently treated and no major side effect was noted (24). Preclinical results of ECT's efficiency have been evaluated on murine models (25). The results are promising with a potentiation of bleomycin and carboplatin.

### Human studies

Several human phase I–II studies have been published, a randomized phase IIB study is actually ongoing, but to date, no preliminary results have been published (26). The main results of clinical studies regarding ECT for the treatment of PDAC are detailed in Table 2. Preliminary results are encouraging with no SAEs and some pain improvement. Available data are however insufficient to assess its effectiveness.

**Table 2.** Electrochemotherapy, clinical trials

References	Patients	Drugs	Electrical settings	Outcome
Granata et al (27)	Clinical phase I–II studies 13 patients with LAPC	Chemotherapy: All patients received systemic chemotherapy prior ECT (gemcitabine + oxaliplatin or 5 FU-irinotecan-oxaliplatin) ECT: Intravenous bleomycin (15,000 UI/m^2^) was injected 8–28 min before ECT	Pancreatic access threw median laparotomy and positioning of 6 peripheric needles around the tumor and 1 central electrode ESOPE protocol: 8–30 PEFs at 910–1,000 V/cm during 100 μs Repeated if needed until the whole lesion and margins were treated PEFs were synchronized with ECG	No serious adverse event occurred No abnormal lipase/amylase elevation was noted Blinded imaging assessment before/after ECT was performed with no significant response according to RECIST Rapid pain reduction was noted
Izzo et al (28)	Prospective phase I–II studies 25 patients with locally advanced PDAC Completes the Granata et al (2015) study			Overall survival of 11.5 mo with better survival when electrodes were manually positioned and adapted to the tumor rather than if they had a predefined geometry (but not significant) 1 mo after ECT, 75% had partial response and 20% stabilization. At 6 mo, 44% and 12%, respectively No SAEs occurred but 8 patients had ascites, 6 pleural effusion, 7 delayed gastric emptying, and 3 splenic infarctions
Casadei et al (29)	Stable patients with LAPC pretreated with chemotherapy + radiotherapy 5 patients	Chemotherapy: 6 cycles before and 3 after ECT with either FOLFIRINOX or gemcitabine + nab-paclitaxel Radiotherapy: 54 Gy ECT: bleomycin 15,000 UI/m^2^ IV	Laparotomic positioning of electrodes (number and position according to tumor) 8 pulses of 1,000 V/cm with clinioporator and ECG synchro complying with ESOPE. Impedance measures were used to adapt the pulses and tension	Reduction in tumor size but not in vascular encasement (no downstaging) No SAEs (3 patients underwent fever) Good quality of life and improvement in pain
Cebron et al (30)	Resectable PDAC (head of pancreas) No neoadjuvant radiochemotherapy 7 patients	ECT: bleomycin 15 mg/m^2^ IV injected 8 min before electroporation	Surgery followed by intraoperative electroporation with 2 plate electrodes of 30 mm separated by 8 mm ESOPE protocol (8 pulses of 100 μs, 960 V, 5 Hz) Several positioning to cover the whole posterior resection area	No AEs related to ECT per or post procedure Several surgical AEs reported (site infection, paralytic ileus, pancreatic fistula, and thrombosis) with 1 SAE Short follow-up but at 6 mo, 3 patients had local recurrence 1 patient with R1 resection had no relapse at 28 mo

AE, adverse event; ECT, electrochemotherapy; ESOPE, European Standard Operating Procedures of Electrochemotherapy; IV, intravenous; LAPC, locally advanced pancreatic cancer; PDAC, pancreatic ductal adenocarcinoma; SAE, serious adverse event.

### Irreversible electroporation

IRE induces cellular death by prolonged nanopore opening in cellular membranes leading to cellular implosion. It offers the advantage of being (almost) nonthermal and to preserve collagen matrix (and thus vascular and biliary structures) compared with other ablative techniques. It is widely used in prostate cancer (Nanoknife).

The results of our systematic review describing its uses for human PDAC treatment are summarized in Table 3.

**Table 3.** Irreversible electroporation, clinical trials

No.	Reference	Patients				Protocol			Outcome						Safety																	Other
		Patients treated with IRE (n)	Age (mean)	Stage	Largest tumor diameter (cm)	Prospective (Y/N)	Pretreatment with chemotherapy (%)	Electrode positioning (PC/SUR)	OS from IRE (median)	IQR	PFS (median)	IQR	OS from diagnosis (median)	IQR	HS (d)	Patients with AEs (n)	SAEs (n)	Pancreatic/biliary fistula	Ascite	Pain	Pleural effusion	Pancreatitis	Thrombosis	Hemorrhage	Delayed gastric emptying	Splenic infarction	Fever	Infection	Digestive perforation	Biliary stricture	Death	
1	Martin et al (31)	54	61	III	NA	Y	91	SUR	17.2	NA	14	NA	NA	NA	NA	32	9	2	3	0	3	0	6	3	2	0	0	6	0	0	0	
2	Martin et al (32)	200	62	III	2.8	Y	100	SUR	24.9	4.9–85	12.4	4.4–38.9	NA		6	74	42	1	0	0	0	1	13	0	0	1	0	18	0	0	3	#1
3	Paiella et al (33)	10	66	III	3	Y	100	SUR	7.5	2.9–15.9	NA	NA	NA	NA	9.5	1	1	1	0	0	0	0	0	0	0	0	0	1	0	0	0	
4	Kluger et al (34)	50	66.5	III	3	Y	100	SUR	12.03	7.7–23.1	9.2	6.6–16.98	NA	NA	6	29	16	2	1	0	0	0	2	6	3	0	0	3	1	1	6	#2
5	Mansson et al (35)	24	65	III	2.75	Y	100	PC	7	4–14	NA	NA	17.9	12–22.4	5	11	3	NA	NA	NA	NA	NA	NA	NA	NA	NA	NA	NA	NA	NA	NA	
6	Narayanan et al (36)	50	62.5	III	3.2	N	100	PC	14.2	9.7–16.2	NA	NA	27	18–36	NA	21	10	0	0	19	0	6	3	0	0	0	0	1	0	0	0	
7	Belfiore et al (37)	29	68	III	5.65	N	100	PC	14	9.9–18.1	17	NA	NA	NA	NA	NA	NA	NA	NA	NA	NA	NA	NA	NA	NA	NA	NA	NA	NA	NA	NA	
8	Scheffer et al (38)	25	61	III	4	Y	100	PC	11	8–17	8	5–10	17	12–19	3	10	2	1	0	3	0	1	0	1	2	0	0	1	0	3	0	#3
9	Lin et al (39)	67	57	III + IV	4.9	N	73	PC							NA	22	0	0	0	17	0	0	0	0	4	0	22	0	0	0	0	#4
	IRE alone	16		III					12.2	NA	7.9	NA	NA	NA																		
	IRE + NK cell	19		III					13.6	NA	9.1	NA	NA	NA																		
	IRE alone	14		IV					9.1	NA	4.8	NA	NA	NA																		
	IRE + NK cell	18		IV					10.2	NA	5.3	NA	NA	NA																		
10	Vogel et al (40)	15	64	III	4.1	Y	100	SUR	16	11–24	NA	NA	NA	NA	NA	8	8	4	0	0	0	0	2	0	3	0	0	0	0	1	0	
11	Huang et al (41)	70	NA	III	NA	N	100	SUR	22.6	19–28	15.4	NA	NA	NA	NA	13	3	1	0	0	0	0	0	1	0	0	0	1	0	0	0	
12	Leen et al (42)	75	63.4	III	3.47	Y	100	PC	27	22–32	15	13.7–16.3	NA	NA	NA	19	1	0	0	15	0	0	0	1	0	0	0	0	0	0	0	#5
13	Liu et al (43)	54	61	III + IV	4.97	Y	NA	PC/SUR							NA	15	4	0	15	6	14	0	1	3	3	0	0	0	0	0	0	
	III + IRE alone	13		III					NA	NA	13.9	8.3–20.2	16.2	13–19																		
	III + IRE + chemo	15		III					NA	NA	16.1	8–21	20.3	16–24																		
	IV + IRE alone	10		IV					NA	NA	9.45	5.3–18.1	11.6	10–13																		
	IV + IRE + chemo	16		iV					NA	NA	11.7	6.6–19.6	13.56	12–20																		
14	He et al (44)	36	NA	III	NA	N	100	PC	NA	NA	7.7	6–10	21.6	NA	NA	23	0	0	1	2	0	0	4	0	0	0	0	0	0	0	0	
15	Mansson et al (45)	24	68	III	NA	Y	100	PC	13.3	11–16	3.9	NA	NA	NA	5	6	6	1	0	0	0	1	0	1	0	0	0	1	0	0	1	
16	Flak et al (46)	33	67.1	III	3	Y	100	PC	10.7	6–30	NA	NA	18.5	13–40	1	21	8	0	2	3	0	0	1	2	0	0	0	3	0	0	2	
17	Holland et al (47)	152	62	III	2.7	Y	100	PC	NA	NA	20.9	16–48	30.3	22–40		27	26	0	0	0	4	4	3	0	0	0	0	4	0	0	3	
18	Ruarus et al (48)	50	61	III + REC	4	Y	100	PC	10	7.5–11	9	6–13	17	12–19	4	29	21	1	0	2	0	3	3	0	3	0	0	3	1	4	2	
19	Ma et al (49)	33	64	III	4.1	Y	100	PC	NA	NA	8.3	6–9	19.8	17–25	NA	3	3	0	2	2	0	2	1	0	1	0	0	O	0	0	0	#6
20	Xu et al (50)	42	58	III	4	N	100	PC							NA	3	2	2	0	0	0	3	1	1	2	0	0	0	0	0	0	
	IRE alone	22							8.6	8–18	NA	NA	NA	NA																		
	IRE + chemotherapy/radiotherapy	20							NA	NA	NA	NA	NA	NA																		#7
21	Yang et al (51)	74	62	III	3.8	Y	100	SUR	NA	NA	28	17–60	38	22–60	NA	13	9	1	8	0	0	0	0	7	11	0	0	1	0	0	0	
22	Veldhuisen et al (52)	30	62	III	3.8	N	100	PC	17.2	10.7–18.9	13.1	9.5–15.3	NA	NA	NA	NA	NA	NA	NA	NA	NA	NA	NA	NA	NA	NA	NA	NA	NA	NA	NA	
23	Pan et al (53)	92	57	III	4.2	Y	NA	PC					NA	NA	7.7	31	NA	0	0	0	0	0	0	1	1	0	31	0	0	0	0	#8
	IRE alone	46							11.8	NA	6.1	NA	NA	NA																		
	IRE + NK cell	46							12.4	NA	7.2	NA	NA	NA																		
24	He et al (54)	64	58.5	III	3.95	N	100	PC	24	18–27	12	8–22	NA	NA	NA	NA	NA	NA	NA	NA	NA	NA	NA	NA	NA	NA	NA	NA	NA	NA	NA	
25	Lin et al (55)	62	62	III	4	Y	84							NA	NA	31	14	NA	NA	NA	NA	NA	NA	NA	NA	NA	NA	NA	NA	NA	0	#9
	IRE + γδ T-cell	30							14.5	10–22	10	8–20	22.5																			
	IRE	32							11	8–15	8	5.5–12	19																			
26	Oikonomou et al (56)	40	65.2	III	3.8	N	83	SUR	NA	NA	10.3	NA	24.2	NA	NA	8	11	8	NA	NA	NA	NA	NA	NA	3	NA	NA	NA	NA	NA	NA	
27	Kwon et al (57)	12	64	III	3.1	N		SUR	13.5	NA	8.6	NA	24.5	NA	NA	NA	NA	NA	NA	NA	NA	NA	NA	NA	NA	NA	NA	NA	NA	NA	1	
28	He et al (58)	85	57.84	III	3.5	N	100	PC							NA	13	NA	13	0	12	0	10	3	0	3	0	0	10	0	0	0	
	IRE + toripalimab	70							NA	NA	NA	NA	44.3	27.5–55																		
	IRE alone	15							NA	NA	NA	NA	23.4	19–30																		
29	Heger et al (59)	25	58	III + REC	NA	N	100	SUR	24	8–28	7	5.8–16	NA	NA	7	20	3	1	6	0	0	2	1	1	0	0	0	20	0	0	0	#10
30	Ma et al (60)	61	64	III	4	N	100	PC							NA	20	4	0	27	39	8	2	0	1	0	0	0	3	0	0	0	
	IRE + gemcitabin	31							17.1	14–20	10.2	9–14	21.5	19–25																		
	IRE alone	30							14.2	11–16	10.2	9–14	16.7	12–22																		
31	He et al (61)	53	57.5	III	NA	N	100	PC	NA	NA	18	NA	28.9	NA	9	10	NA	NA	NA	NA	NA	NA	NA	NA	NA	NA	NA	NA	NA	NA	NA	
32	Thomas et al (62)	100	NA	III	NA	N	100	NA	NA	NA	8.51	4.95–20.2	28.71	19.17–51.19	NA	NA	NA	NA	NA	NA	NA	NA	NA	NA	NA	NA	NA	NA	NA	NA	NA	#11
33	Woeste et al (63)	187	62	III	NA	N	100	SUR	22.4	NA	16.1	NA	25.5	NA	NA	49	48	1	5	0	0	1	0	1	1	0	2	6	0	0	6	
34	Ma et al (64)	103	NA	III	4.1	N	100	PC							NA	80	36	26	12	80	6	34	0	19	0	0	18	0	0	0	0	
	IRE + immunotherapy	25							NA	NA	NA	NA	23.6	18–29.5																		
	IRE alone	78							NA	NA	NA	NA	19.4	12–22																		
35	Martin et al (65)	75	61	III	2.9	Y	100	SUR	24.4	NA	9.4	NA	34.2	NA	NA	NA	NA	NA	NA	NA	NA	NA	NA	NA	NA	NA	NA	NA	NA	NA	NA	#12
36	Tasu et al (66)	17	61	III	NA	Y	100	PC	24.5	NA	NA	NA	NA	NA	NA	10	25	NA	NA	NA	NA	NA	NA	NA	NA	NA	NA	NA	NA	NA	NA	#13
37	Timmer et al (67)	34	65	III	NA	Y	100	PC	12.5	10.9–17	NA	NA	NA	NA	NA	19	8	0	0	0	2	0	0	0	0	0	0	0	0	1	1	#14
38	Zeng et al (68)	38	67.7	IV	4.29	Y	100	PC	14	8–17	6	3.5–10	0	0	5.9	13	4	0	0	13	0	1	0	1	1	0	10	0	0	1	0	#15

AE, adverse event; HS, hospital stay duration; IQR, interquartile range; IRE, irreversible electroporation; n, number; OS, overall survival; PC/SUR, percutaneous/surgical; PFS, progression-free survival from IRE; SAE, severe adverse event; stage III, locally advanced pancreatic cancer (LAPC); stage IV, metastatic pancreatic cancer; Y/N, yes or no.

Other: #1: 50/200 for margin enhancements; #2: 24/50 for margin enhancement; #3: downstaging in 2 patients, local control in 9; #4: better OS among stages III and IV in the IRE + NK group; #5: 4 patients downstaged to be resectable, 3 were R0; #6: OS and PFS with IRE are significantly longer than GEMCITABIN alone; #7: data are not provided as OS; there is an improved survival rate in the IRE + chemotherapy/radiotherapy group; #8: no significant benefit; #9: randomized trial; #10: 11/25 PDAC recurrences; #11: 61/100 for margin enhancement; #12: for margin enhancement; #13: 35% R0-R1 resection after IRE; #14: randomized vs radiotherapy, no significant difference between radiotherapy and IRE; and #15: better OS than chemotherapy alone but unsignificant.

In contrast to ECT, no standard IRE protocol is available. However, a recent expert consensus (69) proposed the following settings: interelectrode distance of 10–20 mm and 90 pulses of 1,500 V/cm with a 90-μs pulse length.

We conducted a statistical analysis of the data collected from the systematic review on IRE (Table 4).

**Table 4.** Statistical analysis: IRE for PDAC treatment

Parameter	Calculation	Value	Population, N ()
Population			2,245
Age (median in years)	Weighted mean	61.7	1,936 (82)
Largest tumoral diameter (median size in cm)		3.6	1,645 (73)
Tumor stage	Total population (% of the population)		
III (LAPC)		2,149 (96)	2,245 (100)
IV (metastatic)		96 (4)	
Study protocol			
Prospective	Total population (% of the population)	1,191 (53)	
Retrospective		1,054 (47)	2,245 (100)
Randomized		96 (4)	
Pretreated with chemotherapy (%)	Weighted mean	98	2,087 (93)
Electrode positioning	Total population (% of the population)		
Surgical		839 (41)	2,056 (92)
Percutaneous		1,217 (59)	
Outcome			
OS from IRE (median)	Weighted mean Weighted median	18.2 17.2	1,495 (67)
OS from diagnosis (median)	Weighted mean Weighted median	26.3 25.7	1,309 (58)
PFS from IRE (median)	Weighted mean Weighted median	12.8 12.2	1,832 (82)
Safety			
Patients experiencing adverse event	Total population (% of the population)		
Studies where it is clearly cited		495 (26)	1,342 (60)
Filling the missing data		699 (36)	1,935 (86)
Severe adverse events		327 (19)	1,705 (76)
Main IRE-related adverse events			1,935 (86)
Pancreatic/biliary fistula		66 (3.4)	
Ascites		82 (4.2)	
Pain		213 (11)	
Pleural effusion		37 (1.9)	
Pancreatitis		71 (3.7)	
Thrombosis		44 (2.3)	
Hemorrhage		50 (2.6)	
Delayed gastric emptying		43 (2.2)	
Splenic infarction		1 (0.1)	
Infection		82 (4.2)	
Biliary stricture		11 (0.6)	
Digestive perforation		2 (0.1)	
Death		25 (1.3)	
Hospital stay (median stay in days)	Weighted mean	6	624 (28)

IRE, irreversible electroporation; LAPC, locally advanced pancreatic cancer.

Population: We included 38 studies with a total amount of 2,245 patients receiving IRE mostly for LAPC (>95%). The median age was 61.7 (n = 1,936). The median tumor size was 3.6 cm. Most of the patients received IRE for tumoral ablation in unresectable tumors and 9% for margin enhancement during surgery.

#### Methodology.

Only 2 studies including 4.3% of the population were randomized. Fifty-three percent of the population came from prospective studies and 47% from retrospective ones. Electrode positioning for IRE was SUR in 41% of the patients and PC in 59%.

#### Safety.

Thirty-six percent of patients experienced AEs, 47% of which severe. AEs occurred more frequently in the PC group (38%) compared with the SUR group (35%), odds ratio 0.82, confidence interval 0.66–1.01, *P* = 0.07. However, there were significantly more SAEs in the SUR group, odds ratio 1.30, confidence interval 1.01–1.7, *P* = 0.04. The most commonly described AEs were pain (30.6% of the AEs), ascites (11.8%), pancreatitis (10.2%), biliary or pancreatic fistulas (9.5%), infections (11.8%), delayed gastric emptying (6.2%), and hemorrhages (7.2). Twenty-five patients died as a direct consequence of IRE (1.3%). The median hospital stay was 6 days.

#### Survival.

**Figure 3.**
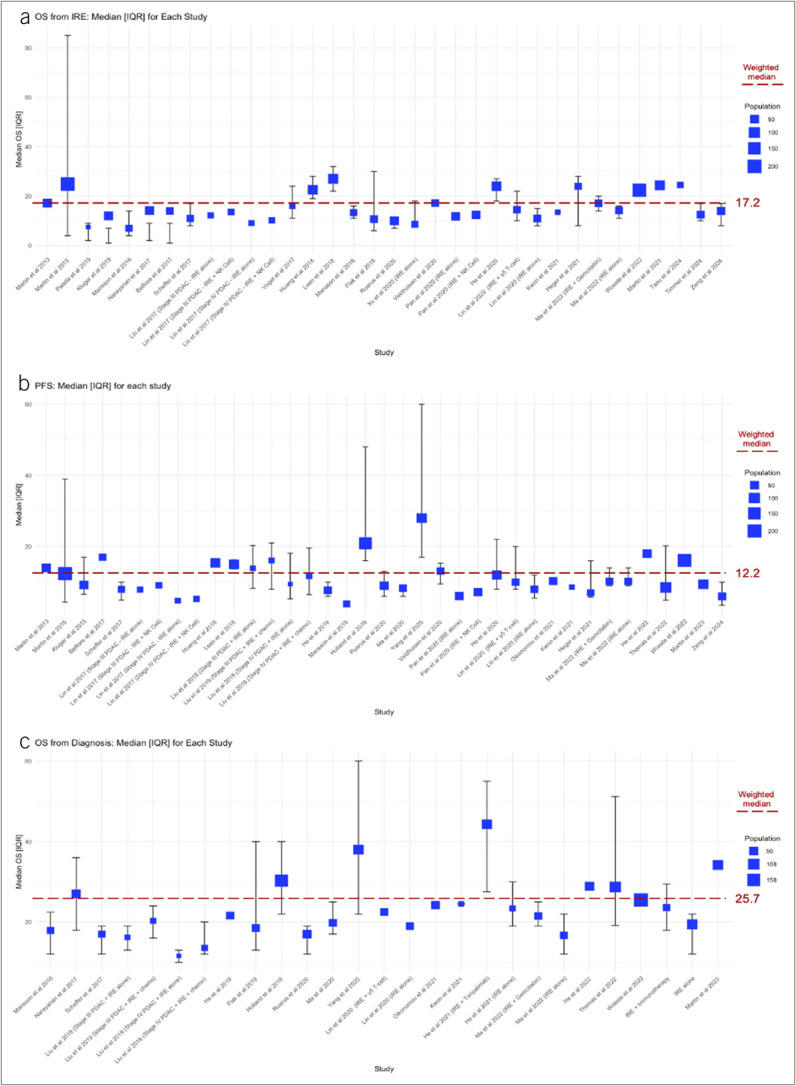
Graphic representation of survival outcomes after IRE for PDAC. IQR, interquartile range; IRE, irreversible electroporation; OS, overall survival; PDAC, pancreatic ductal adenocarcinoma; PFS, progression-free survival.

The estimated weighted median of OS after IRE was 17.2 months, the overall PFS was 12.2 months, and the overall OS from diagnosis was 25.6 months. Figure 3 offers a graphic representation of survival outcomes.

### Electroporation and immunotherapy

Table 5 summarizes the different trials describing a potential benefit of electroporation (reversible or irreversible) on immunotherapy (70). Immunotherapy seemed to improve the survival of patients treated with IRE. To date, no randomized trial has compared IRE + immunotherapy with the standard of care.

**Table 5.** Electroimmunotherapy, preclinical, and clinical trials

References	Model description	Drugs and electrical settings	Outcomes
*In vivo* studies			
Narayanan et al (7)	Immunocompetent mice with subcutaneous injection of PDAC cells from KPC mice	No immunotherapy injection but assessment of the immune response induced by IRE	IRE alone significantly inhibited tumor growth compared with controls and induced systemic adaptive immune response which is promising regarding potential benefits of IRE + immunotherapy
Zhao et al (71)	Murine model with orthotopic PDAC	IRE with anti-PD1 immune checkpoint blockade	Promotes selective tumor infiltration by CD8^+^ T cells Significantly prolongs survival
Human studies			
Lin et al (39)	Unresectable PDAC (stage III, 19 patients; IV, 18 patients)	IRE with allogenic natural killer cell immunotherapy	No major adverse events were described Median progression-free survival and overall survival were higher in IRE + natural killer immunotherapy compared with IRE alone
He et al (58,72)	Unresectable PDAC (stage III, 70 patients)	IRE alone against IRE + toripalimab (antiPD1)	Description of an IRE-based immunomodulatory effect (58) Median overall survival in the IRE + toripalimab arm (n = 15) was 44 mo vs 23 mo in the IRE alone group (n = 70)
Lin et al (55)	Unresectable PDAC (stage III, 30 patients)	IRE alone against IRE + adjunction of γδ-T cell	Improved survival in the immunotherapy arm
Ma et al (64)	Unresectable PDAC (stage III, 25 patients)	IRE alone against IRE with PD1 or PDL1	OS was significantly improved in the immunotherapy arm compared with the IRE arm In multivariate analysis, immunotherapy was an independent factor for improved survival

IRE, irreversible electroporation; PDAC, pancreatic ductal adenocarcinoma.

## DISCUSSION

### Electrochemotherapy

ECT has shown promising results in *in vitro* and murine models of PDAC, although we have access to a limited number of studies. The most commonly used drug was bleomycin with an electrical protocol following the ESOPE consensus.

Whereas several works have proven the benefits of ECT in oncology, few studies in patients with PDAC are available to date. One randomized trial comparing ECT with bleomycin with standard of care is currently ongoing with no published results to date.

Further preclinical studies on innovative translational models such as tumor slices or murine immunocompetent models could help investigate the value of combining ECT and immunotherapy.

One major limitation for the human applications of ECT and IRE is the difficulty of the pancreatic access (needing to be radiological or even surgical). This difficulty increases the morbidity of procedures and limits its recurrences to 1 single application. Minimally invasive approaches, such as endoscopically guided devices, could provide safer and repeatable means to perform pancreatic ECT.

We can conclude from the available data that the outcomes of ECT are promising on pain management, but further studies are needed to explore the OS or PFS benefits. ECT seems safe compared with the AEs described in the IRE, or other ablative procedures but larger studies have to be performed to confirm this trend.

### Irreversible electroporation

#### Benefits of IRE.

We present, to our knowledge, the largest literature review regarding IRE in PDAC (73–75). Most of the patients had LAPC. The OS from IRE was 18.2 months. Among the studies described, few compared IRE with standard of care and none with a proper randomized design. The only available randomized studies compared IRE with radiotherapy (with no significant difference) or with IRE with immunotherapy, showing in that case some benefit of the adjunction of immunotherapy.

When comparing the calculated OS after IRE with the reported survival of LAPC with folfirinox (17.1 months) (76) or with OS from patients who do not undergo surgical resection after chemotherapy (16.3 months) (77), IRE does not seem to extend survival significantly. This trend is confirmed by a recent meta-analysis highlighting the absence of significant survival benefits of IRE (75). A randomized trial is actually ongoing (PMC11662571), but the lack of significant benefit of IRE associated with a high AE rate pleads against its use in clinical practice.

#### Side effects.

About one-third of the treated population experimented side effects with 47% of those being classified as severe, including intra-abdominal hemorrhage, fistulas, or pancreatitis and with an overall IRE-related mortality exceeding 1%. These results are consistent with other systematic reviews (78). There was no significant association between AEs and the SUR or PC approach; however, the SUR approach was significantly associated with a higher number of SAEs. It is noteworthy that the procedure is contraindicated if a self-expandable biliary stent is present in the electric field, which is a particularly common occurrence in PDAC management (79). No mentions were found regarding the risks of RE if a biliary stent is positioned, but the few available clinical studies included patients with biliary stents and no major AE occurred suggesting that the inference of the metallic stents might be related to the higher current delivered during IRE. This limitation should however be taken into consideration, should RE and ECT be used in common practice.

#### IRE compared with other ablative techniques.

In contrast to ECT and electroimmunotherapy, IRE is an ablative technique and can be compared with other minimally invasive procedures such as RFA, microwave ablation (80), brachytherapy (81), or cryotherapy (82,83). If the data regarding the latest remain scarce, several studies describe the pancreatic uses of RFA which be performed under endoscopic-ultrasound guidance. For PDAC treatment (84), RFA is followed by approximately 13% of AEs with some benefits on tumor growth or pain management but no demonstrated survival benefit (85,86). RFA however progressively integrates the common practice with its applications for small (<2 cm) pancreatic neuroendocrine tumors such as insulinomas (87).

#### Limitations and bias.

The conclusions provided by this literature review on IRE are subject to limitations.

##### Population.

Patients recruited in the studies included for this IRE review were hyperselected. They were also relatively young compared with the literature (88) but had a tumor size comparable with other cohorts of LAPC (77). Our review is also subject to publication bias that could lead to overlook negative survival and/or morbidity results.

##### Methodology.

Many studies are retrospective or stemming from prospectively maintained databases. Some teams published successive cohorts but with overlapping inclusion times which means that some patient's data might be analyzed twice.

##### Data analysis.

Several missing data were reported which leads to bias. In several studies, the number of patients experiencing AEs was not described. We estimated that it was at least equal to the most described AEs. This allowed to include more studies in the AEs analysis but can underestimate the total amount of AEs. For SAEs however, calculations were made only on available data because it was not possible to infer if not specified. Finally, the available data did not allow to perform multivariate analysis. Our results remain however consistent with other literature reviews (75).

### Immunotherapy and electroporation

Immunotherapy has significantly improved therapeutic outcomes in some cancers (skin, lung, and stomach), but efficacy results have been elusive in PDAC so far. Among others, one reason could be the dense and immunosuppressive TME surrounding PDACs limiting cytotoxic lymphocyte trafficking. The abscopal (literally, “away from target”) effect describes the action on metastasis of the treatment of a local lesion with an ablative therapy which is supposed to rely on the release of tumoral antigens after local ablation, enhancing global antitumoral immunity (89–91). Some preclinical works on IRE highlight similar effects with an increase of regulatory T cells and PD-1^+^ T cells after IRE on small groups of human patients (92).

A synergy between ECT and immunotherapy has been described for melanoma since 2003 (93). Studies have demonstrated a higher efficiency in melanoma of ipilimumab (antiCTLA-4) + ECT (94) and pembrolizumab (antiPD1) + ECT (95) vs immunotherapy alone. Few studies have described the association of immunotherapy and electroporation, but to date, no study has focused on ECT + immunotherapy combination. Some IRE + immunotherapy studies were included in the analysis and seemed to provide a better OS compared with IRE alone. The adjunction of immunotherapy was not associated with significantly higher AEs/SAEs rates in these studies. These results are promising and deserve further investigation because other ablative techniques such as radiotherapy are not associated with such a strong potentiation (96).

Electroporation for the treatment of pancreatic cancer has been studied for the past 20 years. IRE to treat locally advanced PDAC does not seem efficient enough, regarding its high rate of AEs to be used in clinical practice. The benefits of combining IRE with immunotherapy are promising but remain experimental and deserve prospective controlled studies. ECT for PDAC is still in its infancy, but its low associated morbidity and potentialities for overcoming the TME barrier and stimulating the immune response bear some promises.

## CONFLICTS OF INTEREST

**Guarantor of the article:** Gabriel Marcellier.

**Specific author contributions:** G.M.: data collection, writing, reviewing, and statistical analysis. T.L., P.R., and M.R.: reviewing. F.P.: reviewing, data collection, and writing.

**Financial support:** None to report.

**Potential competing interests:** None to report.

**Availability of data:** The complete data collected are included in the study tables.

**Acknowledegments**: We could like to express our gratitude to the teams involved in the PACTE projetct (Pancreatic Cancer Treatment using EUS-Guided Electroporation), as well as to the members of our research consortium (APHP, Ampère, LGEF, ILM) for their invaluable collaboration and support throughout this study.

## ABBREVIATIONS:

AE: adverse event
CT: computed tomography
ECT: electrochemotherapy
HS: hospital stay
IC50: inhibitor drug concentration
IQR: interquartile range
IRE: irreversible electroporation
LAPC: locally advanced pancreatic cancer
NA: not available
OS: overall survival
PC: percutaneous
PDAC: pancreatic ductal adenocarcinoma
PEF: pulsed electric field
PFS: progression-free survival
RE: reversible electroporation
RFA: radiofrequency ablation
SAE: severe adverse event
SUR: surgical
TME: tumor microenvironment
